# Segmentation Scale Effect Analysis in the Object-Oriented Method of High-Spatial-Resolution Image Classification

**DOI:** 10.3390/s21237935

**Published:** 2021-11-28

**Authors:** Shuang Hao, Yuhuan Cui, Jie Wang

**Affiliations:** 1 School of Natural Science, Anhui Agricultural University, Hefei 230036, China; cuiyh@ahau.edu.cn; 2 School of Resources and Environmental Engineering, Anhui University, Hefei 230039, China; wangjie@ahu.edu.cn

**Keywords:** scale, OBIA, Worldview-3, CART model

## Abstract

High-spatial-resolution images play an important role in land cover classification, and object-based image analysis (OBIA) presents a good method of processing high-spatial-resolution images. Segmentation, as the most important premise of OBIA, significantly affects the image classification and target recognition results. However, scale selection for image segmentation is difficult and complicated for OBIA. The main challenge in image segmentation is the selection of the optimal segmentation parameters and an algorithm that can effectively extract the image information. This paper presents an approach that can effectively select an optimal segmentation scale based on land object average areas. First, 20 different segmentation scales were used for image segmentation. Next, the classification and regression tree model (CART) was used for image classification based on 20 different segmentation results, where four types of features were calculated and used, including image spectral bands value, texture value, vegetation indices, and spatial feature indices, respectively. WorldView-3 images were used as the experimental data to verify the validity of the proposed method for the selection of the optimal segmentation scale parameter. In order to decide the effect of the segmentation scale on the object area level, the average areas of different land objects were estimated based on the classification results. Experiments based on the multi-scale segmentation scale testify to the validity of the land object’s average area-based method for the selection of optimal segmentation scale parameters. The study results indicated that segmentation scales are strongly correlated with an object’s average area, and thus, the optimal segmentation scale of every land object can be obtained. In this regard, we conclude that the area-based segmentation scale selection method is suitable to determine optimal segmentation parameters for different land objects. We hope the segmentation scale selection method used in this study can be further extended and used for different image segmentation algorithms.

## 1. Introduction

As the most efficient method of obtaining the land surface information in regional areas [1,2], space-based optical remote sensing devices have been significantly improved in the last few decades. The remote sensing image analysis methods based on pixels have promoted the continuous progress of image analysis techniques and theory. Supervised and unsupervised classification [3,4,5] methods have been widely applied to the extraction of remote sensing image information. Machine learning algorithms, including the support vector machine, random forest, and artificial neural network algorithms, have also been developed, along with the techniques and theory [6,7,8]. As Earth observation via satellites has been rapidly developed in recent years, the capability for multiple spectral image data collection has been continuously enhanced, and thus, a variety of high-resolution image types have been collected using different sensors [9,10]. This is a large step forward in the application of remote sensing technology. However, the tremendous amount of high-resolution image data also imposes new requirements and challenges in image processing techniques and theory. Since many different types of high-spatial-resolution images are used in a variety of remote sensing research fields, the traditional image classification algorithms cannot efficiently solve the problem of high-spatial-resolution images. This has encouraged researchers to invest more effort in the development of methods of processing high-resolution remote sensing images that contain massive amounts of information about land objects.

Since high-spatial-resolution images contain a more complicated spectrum range, more detailed information about the land objects can be obtained, which can enhance land objects’ spectral differences [11]. Especially for land objects in areas with complex feature categories, pixels can no longer be the only factor considered in image analysis and information extraction and the land objects’ spatial domain differences should also be taken into consideration. As the spatial resolution increases, the spectral heterogeneity of the same object is also enhanced, which means detailed information about the land object’s spectral features and textural features is more complicated, and even the comparison of different land objects’ geometric structures is more obvious. As spatial resolution has improved, the amount of data has grown exponentially, and how to efficiently extract information from high-spatial-resolution remote sensing images has become one of the main challenges for researchers.

Traditional remote sensing image classification is based on pixels. This type of method extracts land objects based only on the image’s spectral information, and it detects the land object at the unit of a pixel. No other features of the land object have been used in information extraction. However, for high-spatial-resolution images, different land objects’ spectral performances may be similar. It is actually not easy for traditional classification methods to distinguish between different land objects with similar spectral information. 

Object-based image analysis (OBIA) is a method of processing high-resolution images [12,13,14,15,16,17]. OBIA divides the image into several coessential but non-overlapping regions, and the regions are processed as objects. These coessential image regions are used as the basic unit in subsequent processing. This method can fully use the feature statistics of the different objects. The related features of different land objects, including spectral, context, and shape features, are extracted for the different land objects [18]. OBIA has been widely used in remote sensing image analysis [19,20,21,22,23,24]. The basic processing unit of the OBIA method is the object, which differs from methods based on the object’s pixels [25], and thus, OBIA has been determined to have a great uncertainty in terms of image processing. 

The uncertainty of OBIA has also caused many problems, including segmentation scale selection, sample training, and accuracy assessment. The segmentation scale affects the accuracy of the image information extraction. A large segmentation scale cannot precisely extract relatively small objects, which means these small objects are submerged by the large objects. When the segmentation scale is small, the large object targets may be over-segmented, which can cause tattered results, and thus, the objects’ features cannot be exactly reflected. The image segmentation scale is one of the most important factors in image analysis and calculation [26,27,28,29,30,31]. Without good segmentation results, excellent information extraction and target recognition cannot be achieved. Therefore, in this study, the land objects’ extraction results based on different segmentation scales were compared, and the scale effect caused by multiresolution segmentation was also analyzed. Therefore, this study aims to explore segmentation scale effect on high-spatial resolution images based on OBIA. First, 20 different segmentation scales were applied in the image segmentation. The CART, which is widely used as the basis for machine learning algorithms, including bagging decision and boosting decision trees [32], has been applied for the image classification in this study. In order to classify the WorldView-3 (WV-3) image using the OBIA method, a framework based on feature selection and multiple segmentation scale comparison was established. Field sampling data and forestry inventory data were used as training and validation samples; the manual interpretation is also applied to the WV-3 image to build the classification framework and prior knowledge. Then, the CART classifier was used to classify the image of the study area, and a confusion matrix was used for the accuracy assessment. Furthermore, an equation that reflects the relationship between the optimal segmentation scale and the land object’s average areas was established, which helped determine the optimal segmentation scale from the perspective of the area-based method. The study result was designed to be incorporated into a workable and trusted technique solution for image classification; it provides an effective method for high-spatial-resolution image classification based on OBIA.

## 2. Materials and Methods

### 2.1. Study Area

**Figure 1:**
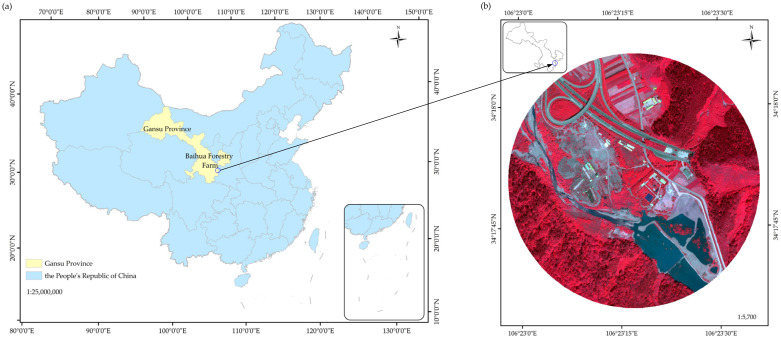
(**a**) Location of the study area; and (**b**) WorldView-3 image of the study area.

The Baihua forestry farm (34°16′–34°25′ N, 106°15′–106°30′ E), which has a total area about 291.02 km^2^, is located in the eastern part of Gansu Province, China (Figure 1a). This study was performed in the southern part of the forestry farm. The Baihua forestry farm is an important water conservancy region in the Jialing River basin. The climate in this region is a warm temperate continental monsoon climate, with an annual average temperature of 10.9 °C. The forest cover rate is about 92.8%. Due to the suitable environment and climate, wild animal and plant resources are abundant, and diverse plant communities can be found in this region. Different types of land cover occur in the study region, of which forest land and shrub grassland are the main types of land cover. Both of these types of land cover account for more than 30% of the total area. The rest of the land is covered by water, roads, barren land, and buildings, each accounting for 5% of the study region. The other land objects accounted for less than 5% and were not considered in the image classification. Thus, according to the main land cover types and field investigations, the classification framework was composed of forest, shrub-grass, barren land, water, roads, and buildings in this study.

### 2.2. Data Resource and Processing

The WorldView-3 sensor was launched by DigitalGlobe Inc. (Westminster, CO, USA) on 13 August 2014. It has the best performance in terms of radiation, spectral, and spatial levels of all of the multispectral satellite sensors currently in orbit [33]. It has one panchromatic and eight multispectral bands in the visible-near infrared (VNIR) region. In addition to the VNIR spectral region, there are also 8 bands in the short-wave infrared (SWIR) region and 12 clouds, aerosols, vapors, ice, and snow (CAVIS) bands, with pixel sizes of 0.31 m, 1.24 m, 3.7 m, and 30 m, respectively [34]. The WV-3 used in this study were acquired on 3 June 2019 (Figure 1b). The multispectral data contains eight bands, including the coastal blue (427 nm), blue (482 nm), green (547 nm), yellow (604 nm), red (660 nm), red edge (723 nm), NIR1 (824 nm), and NIR2 (914 nm) bands. The original WV-3 images were delivered at the 2A-level, and in order to meet the processing requirement, the images were processed using the ENVI software. The processing included radiometric calibration and orthorectification; after the preprocessing, the Gramm–Schmidt method was used to merge the multispectral image with the panchromatic image in order to obtain a pansharpened image with a resolution of 0.3 m.

### 2.3. Image Segmentation

By segmentation processing the image would be segmented into a spatially contiguous objects set, every object in the set is composed of a certain number of pixels with homogeneity [17]. The multiresolution algorithm (MRS) was used for image segmentation; this algorithm is widely used in image segmentation studies [35,36,37]. The MRS algorithm is complex and has high demands of its users. Users have to customize different parameters, which include scale, shape, or compactness [38]. The basic principle of MRS is that it comprehensively considers the spectral features and shape features of multispectral images and uses the bottom-up iterative merging algorithm to segment the images into highly homogeneous patch objects [26,39]. 

Segmentation is the basis of and is crucial for OBIA; it significantly impacts classification accuracy. Different pixels with a high inner homogeneity are divided into object classes, while the adjacent objects exhibit significant dissimilarity. The object’s homogeneity can be evaluated based on the standard deviation of the pixel value inside the object, while the heterogeneity can be identified by the object’s spectral heterogeneity, shape heterogeneity, or texture heterogeneity. When the object’s attribute exceeds the heterogeneity threshold defined by the segmentation scale, the growth of the object stops. After optimization, the internal weighted heterogeneity of each object is minimized. The segmentation object’s heterogeneity (*H*) contains the spectral heterogeneity and the shape heterogeneity, and the calculation equations are as follows [40]:

(1) $$H=\omega_{color}h_{color}+\omega_{shape}h_{shape}$$

(2) $$h_{shape}=\omega_{compact}h_{compact}+\omega_{smooth}h_{smooth}$$

where *ω_color_* is the spectral weight, *ω_shape_* is the shape weight, and *ω_color_* + *ω_shape_* = 1; *h_shape_* is the shape heterogeneity, which is composed of *h_compact_* and *h_smooth_*; *ω_compact_* is the compact weight, *ω_smooth_* is the smooth weight, and *ω_compact_* + *ω_smooth_* = 1.

As a bottom-up algorithm, the MRS treated each individual pixel as a separated object at the beginning of the procedure. When the segmentation is processed, two optimal image objects are selected and merged. They are selected and merged based on the change of heterogeneity [16,41], which can be explained based on the following equation:

(3) $$h_{diff}=\left(s_{a}+s_{b}\right)h_{merge}-\left(s_{a}h_{a}+s_{b}h_{b}\right)$$

where *h_a_* and *h_b_* are the heterogeneity of two original objects, *s_a_* and *s_b_* are the original object’s size, and *h_merge_* is the heterogeneity of the merged object. By limiting the change of the heterogeneity within a reasonable range, the objects with great variance are not merged. Therefore, the merged objects should not change the heterogeneity significantly. The heterogeneity changing is related to the segmentation scales; to obtain good merging results, the change of the heterogeneity should not be larger than the scale parameter.

### 2.4. Feature Selection and Extraction

The image features play an important role in information extraction and change detection. In this study, the objects’ multispectral, textural, and spatial features were used as multiple features to classify the WV-3 image. The multispectral indices and textural feature were computed based on the eight bands of the WV-3 image described above. Each of the eight bands’ reflectance value data were also used as a single feature for the image classification. The spectral bands’ maximum differences and brightness were also calculated and used as features. In addition to the multiple spectral origin, the Normalized Difference Vegetation Index (NDVI) [42], Ratio Vegetation Index (RVI) [43], Difference Vegetation Index (DVI) [44], and Normalized Difference Water Index (NDWI) [45] were used as potential features for the classification. The features are shown in Table 1.

The textural features are formed by a large number of repeated occurrences of small objects in the image. They are a comprehensive reflection of the individual object’s size, shape, shadow, and color, and they describe the spatial variation characteristics of the pixel brightness. Previous studies have demonstrated that textural features can serve as an effective supplement for the spectral information of high-resolution images, and they can also improve the accuracy of classification and information extraction [46,47,48]. In order to enhance the land object information, the texture of the WV-3 image was computed based on the gray level co-occurrence matrix (GLCM). After the WV-3 images were segmented, based on the eight spectral bands, the textural features were calculated for the image objects.

The GLCM algorithm can describe the image brightness, adjacent space, and change range. It is the second-order statistical feature of the change in the image’s brightness. If the size of two-dimensional digital data *f*(*x*, *y*) is *M* × *N* and the gray level is *N_g_*, then the GLCM can be expressed as follows [49]:

(4) $$P(i,j)=E\left\{(x_{1},y_{1}),(x_{2},y_{2})\in M\times N|f(x_{1},y_{1})=i,\;(x_{2},y_{2})=j\right\}$$

where *P* is an *N_g_* × *N_g_* matrix, and *E*(*x*) is the element number in set *X*. If distance between (*x*_1_, *y*_1_) and (*x*_2_, *y*_2_) is *d*, and the included angle with the abscissa axis is *θ*, then *p* value (*i*, *j*, *d*, *θ*) of the different distances and angles can be calculated. The measures of the texture used in this study were homogeneity, contrast, dissimilarity, entropy, standard deviation, correlation, angular 2nd moment, and mean. 

In addition to the spectral and textural features, the spatial features were also used in the image classification. The spatial features include area index, density index, rectangular fit, elliptic fit, asymmetry, compactness, border index, and shape index. Overall, there are 66 different features for the three types of features described above. The equations for calculating the selected textural features are presented in Table 2.

### 2.5. CART Algorithm

The basic concept of decision tree analysis is splitting the dataset into increasingly homogenous subsets. The decision tree algorithms are a typical non-parametric method and can be easy to interpret, so these algorithms are widely used in image classification [51,52,53]. As a non-parametric algorithm, the classification and regression tree model (CART) is resistant to missing data and a normal distribution of the variables is not strictly required [54]. The CART model cycle analyzes the training dataset to form a binary tree, of which, the training dataset is established using the test variables and target variables. As a supervised classification method, the CART model also needs to establish and evaluate the CART based on specific training samples before classification. The model uses the following method to learn the training samples:

(5) $$\begin{array}{l} L:=\left\{X_{1},X_{2},\cdots ,X_{m},Y\right\}\\ X_{1}:=\left(x_{11},x_{12},\cdots x_{1t1}\right)\\ \ldots \\ X_{m}:=\left(x_{m1},x_{m2},\cdots x_{mtn}\right)\\ Y:=\left(Y_{1},Y_{2},\cdots ,Y_{k}\right) \end{array}$$

where the *X*_1_, *X*_2_, …, *X_m_* are the attribute vectors, and *Y* is the label vectors. Their property format is not strictly regulated, and it can be sequenced or discrete. 

The *Gini* index is used as the principle to select the best testing variable and segmentation threshold. The index is used to measure the data division and the impurity of the training dataset. A lower *Gini* index means that the sample’s purity is high, and it can also indicate that the probability of the samples belonging to the same category is high. The *Gini* index is defined as follows:

(6) $$Gini=1-\sum_{i=1}^{m}P_{i}^{2}$$

where *P* is the probability that the element belongs to the *i*^th^ category. The *Gini* index, as a measure of the heterogeneity, was used as the splitting rule [50].

One or more attribute sets are selected by the CART model from multiple predicted attributes, and the selected sets are set as the split variable of the tree node; then, the test variables are sent to different branches. A large tree is established by repeating the process, and the pruning algorithm is used to optimize the generated results. The CART algorithm is suitable for a wide range of image mapping and predicting problems [54,55]. As a widely used machine learning algorithm, the CART is sensitive to the training samples, and in order to obtain ideal classification results, the training samples should be representative of broad sections of the land objects. In this study, the training samples of the different land objects were randomly selected, and the training samples were uniformly distributed in the study area.

### 2.6. Accuracy Assessment

The field sampling data were used as training and validation samples. There are 90 individual field sampling spots of 5 different land objects. Due to the field sampling, data are limited, and in addition to these field sampling data, the forestry resource inventory data were also randomly selected by ArcGIS. In total, there are 1000 points selected. Based on the selected sample points, the error matrix was established to evaluate the results. Of the random points, 20% were selected and set as the independent sample to validate the classification result, and the user’s accuracy (UA), producer’s accuracy (PA), and the overall accuracy (OA) were calculated based on the confusion matrix. The precision, recall, and area under curve (AUC) were also used as the evaluating indicator to verify the efficiency of the algorithm.

**Figure 2:**
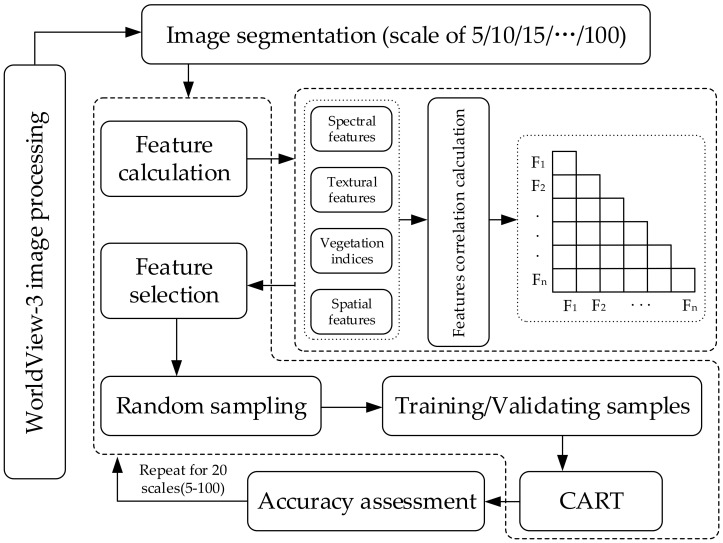
Flowchart of the study process.

The general flowchart of the study process (Figure 2) was as follows.

## 3. Results

### 3.1. Image Segmentation

**Figure 3:**
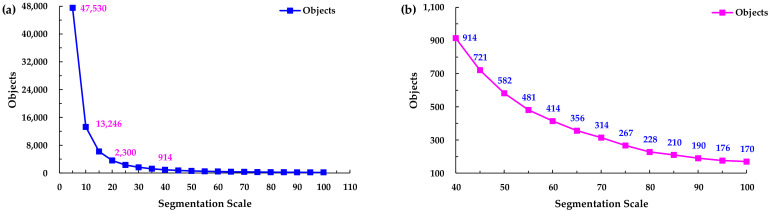
Scale versus number of objects. (**a**) General view of segmentation scale 5–100; (**b**) Local enlarged view of segmentation scale 40–100.

Different segmentation scales significantly affect the classification results, so in order to examine the effect of the scale on the information extraction ability, different segmentation scales were used to segment the WV-3 image in this study. Multiple segmentation scales from 5 to 100, with a step size of 5, were used. Thus, the image was processed and analyzed using different segmentation scales. The effects of the color, shape, smoothness, and compactness were not considered in this study, so every segmentation scale shared the same weights for color (0.9), shape (0.1), smoothness (0.5), and compactness (0.5). Based on the segmentation results, it was determined that a small segmentation scale causes over-segmentation of the image, which means excessive polygon objects will be generated, and thus, the same object may be divided into different categories. Over-segmentation is detrimental to selecting the training samples. For a large segmentation scale, the heterogeneity will increase during the image classification process. The ideal segmentation scale will obtain a result that can show the differences between different blocks, but the internal homogeneity is high. The different segmentation scales resulted in different numbers of objects being identified (Figure 3). As can be seen from Figure 3, the number of segmented objects decreased rapidly from a scale of 5 to a scale of 40, but when the scale was between 40 and 100, the trend of the change in the number of objects became stable. 

### 3.2. Feature Selection and Analysis

As the segmentation scale changed, the spectral, textural, and spatial features involved in the CART algorithm were different, and not every feature mentioned above was used in the classification. For instance, the costal blue feature was selected 19 times for the classification, which is the highest of all of the 66 alternate features, while 12 of the features were not used in the classification for any of the segmentation scales, including angular 2nd moment (90° and 135°), contrast (135°), dissimilarity (0°), entropy (all directions and 90°), homogeneity (all directions), mean (45°), standard deviation (0° and 90°), NDWI2, and RVI1.

According to the results, for the spectral features, the change trends of relatively important features at different segmentation scales are basically the same. For all 20 segmentation scales, the coastal blue feature was selected 19 times, and only scale 45 did not use coastal blue as a classification feature. Scale 5 selected 7 spectral features to extract the information, which was the most of all the segmentation scales. For textural information, dissimilarity 90° was selected seven times. Scale 40 selected 7 textural features to extract the information, which was the most of all the segmentation scales. For the spatial features, the density and border index were selected 11 and 9 times, respectively. Scale 70 selected 4 spatial features to extract the information, which was the most of all the segmentation scales. According to the results, the vegetable indices were not as important as the spectral, textural, and spatial features. Especially for the large segmentation scales, hardly any vegetation indices were selected for information extraction. Overall, 15 features were selected by scales 40 and 70, which were the largest numbers of features involved in the image classification, while only 7 features were involved in the image classification for scale 100. 

**Figure 4:**
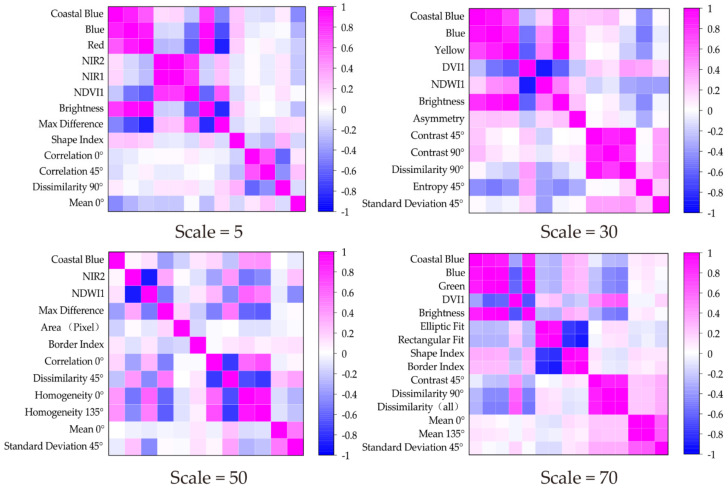
Segmentation objects’ feature correlations for different segmentation scales.

The different features have different levels of importance to image classification. In order to analyze the features’ correlations at the different segmentation scales, the Pearson correlation was calculated. Figure 4 shows the features’ correlations at different segmentation scales (Scale = 5, 10, 30, 70). There are three main colors, magenta (positive correlation), blue (negative correlation), and white (irrelevant), and the correlation coefficient increases as the color becomes darker. According to the correlation analysis results, the spectral features tend to be positively correlated. Most of the textural feature for the small segmentation scales (5–35) are positively correlated, but as the segmentation scale increases (40–100), although they remain strongly correlated, the number of negatively correlated features increases. The spatial features are more strongly correlated than the other three types of features. Overall, positive correlations are more widespread in the selected features. For scales 15 and 80, both selected 11 different features for the information extraction, but according to the correlation images, the spectral features’ correlations are higher at small segmentation scales than at large segmentation scales. For scale 15, the spectral features’ correlation coefficients are coastal blue/blue = 0.89, blue/yellow = 0.89, and coastal blue/yellow = 0.74, while the correlation coefficients of the spectral features for scale 80 are coastal blue/blue = 0.94, coastal blue/NIR1 = −0.0097, coastal blue/NIR2 = −0.0018, blue/NIR1 = −0.28, and blue/NIR2 = −0.29. According to the correlation calculation results, the strong positive correlations are mostly concentrated in the upper left corner, which means most of the spectral features and spatial features are autocorrelated. However, when checking the lower right corner, it was found that the spectral and textural features are correlated. Some of the features’ correlations change as the scale changes. Both scales 5 and 80 have coastal blue and dissimilarity 90°, but their correlations changed. For scale 5, the correlation between coastal blue and dissimilarity 90° is 0.086; however, for scale 80, the correlation is -0.272. Therefore, as the segmentation scale increased, their correlation decreased.

According to the results, the spectral features play an important role in the image classification, and their information advantages are obvious. For scales of 40–70, the textural features account for a relatively larger proportion than the other types of features. The spatial features were selected at every segmentation scale in this study, but they accounted for a relatively small proportion. For the vegetation indices, Figure 4 shows that they are not as important as the spectral, textural, and spatial features. Especially for the large segmentation scales, no vegetation indices were selected as valuable features for the OBIA method of image classification, which indicates that the vegetation indices used in this study were correlated with the spectral features. Based on the above analysis and Figure 4, for the OBIA method, the spectral features still play an important role in information extraction. Several previous studies [50,56] have demonstrated that the textural features can effectively improve the OBIA method of classification accuracy. However, although texture calculation requires a large computation capacity and time, by increasing the classification’s accuracy, it can also decrease the efficiency of OBIA image classification. Thus, when attempting to increase the efficiency of the information extraction, the textural features are not the best choice, and the spectral features are irreplaceable.

### 3.3. Object Areas and Segmentation Scale Relationship Analysis

**Figure 5:**
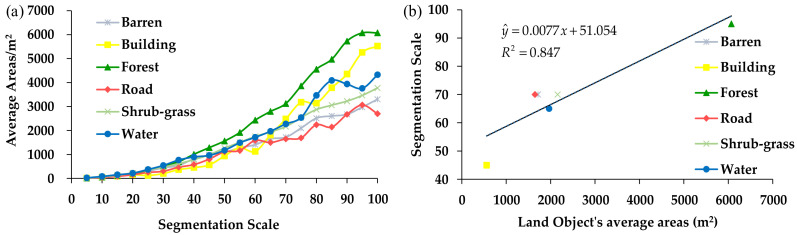
Relationship between the optimal segmentation scale (**a**) and the land objects’ average areas (**b**).

The classification results for the different segmentation scales indicate that the different land objects’ optimal segmentation scales are different. Of the six types of land objects, the average forest areas increased fastest as the segmentation scale increased. According to Figure 5a, for segmentation scales of 5–60, the growth rate of the building area was the slowest, and when the segmentation scale was >80, the growth rate of the building area was the second highest. The growth rate of the barren land was consistent with the grow trend of the shrub-grass land. In order to discuss the relationship between the land objects’ areas and the segmentation scale, the average areas of different land objects were calculated to analyze their relationships with the segmentation scale. The analysis results show that the land objects’ areas are strongly correlated with the segmentation scale. The correlation coefficient *R*^2^ between the land objects’ average areas and their optimal segmentation scales is 0.847 and *p*-value is 0.015 < 0.05, which means the land object’s ideal area is significantly affected by the segmentation scale. The equation shown in Figure 5b is $\hat{y}=0.0077x+51.024$, where *y* is the segmentation scale, and x is the average area of the land object in the study region. For the WV-3 image used in this study, this equation indicates that the different land object’s optimal segmentation scale can be determined according to the land object’s average areas for the OBIA method [14]. For instance, if the average area of Barren is 1000 m^2^, the optimal segmentation scale for the barren land in the WV-3 image would be about 60 based on the above equation.

### 3.4. Classification Results

The WV-3 image was segmented based on the principle of the OBIA algorithm; the extracted features are used as training data set and the CART classifier based on the Gini index is trained. The Gini index is a measurement of heterogeneity. It was used as the splitting rule. The image segmentation and segmented object’s different feature were calculated under eCognition software. The precision, recall, and AUC were calculated to validate the CART model, and the overall accuracies were also calculated based on confusion matrices. The value of these evaluating indicators are shown in Table 3.

According to the relationship between the segmentation scale and the land objects’ areas, the optimal segmentation scale of the three types of land objects (i.e., shrub-grass, roads, and barren land) is 70. The optimal segmentation scales for forest, buildings, and water are 95, 45, and 65, respectively. The different land objects have different optimal segmentation scales. According to the study results, the forest areas account for about 34% of the study area, while the shrub-grass and barren land areas account for about 30% and 16% of the study area, respectively. Overall, the optimal segmentation scale of the study area may be within the range of 45–70. Based on the above analysis, the WV-3 image was processed at a segmentation scale of 45–70, with a step size of 5.

**Figure 6:**
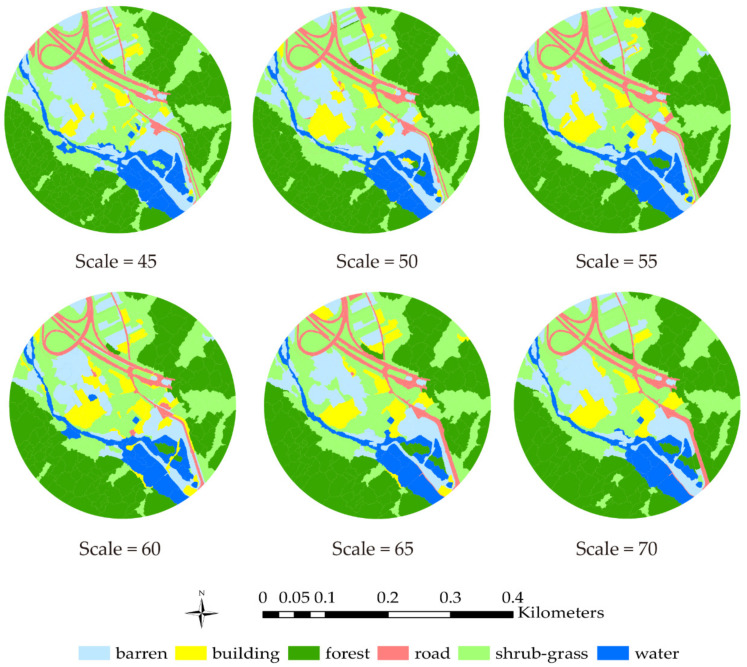
Classification results for segmentation scales 45–70.

Based on the calculation results, it is obvious that the CART algorithm and model are capable of land cover classification and prediction. Based on accuracy assessment results, at the optimal segmentation scale of 45–60, the relative evaluating indicator value decreased as the segmentation scale increased. The AUC of segmentation scale 70 is 0.9182, which is the lowest of the optimal segmentation scale range. For the different land objects obtained based on scale 70, the precision of road and building areas were 0.5, which were the lowest of all the land cover types. The road and building object features are not as obvious as the other four kinds of land cover types, which might cause relatively lower classification and prediction accuracy. The image features of road and building areas are similar. Since the segmentation scale is relatively large, the segmented object might contain different land objects; distinguishing a road or building from other land objects is not easy at segmentation scale 70. The classification results (scale of 45–70) are shown in Figure 6. 

**Figure 7:**
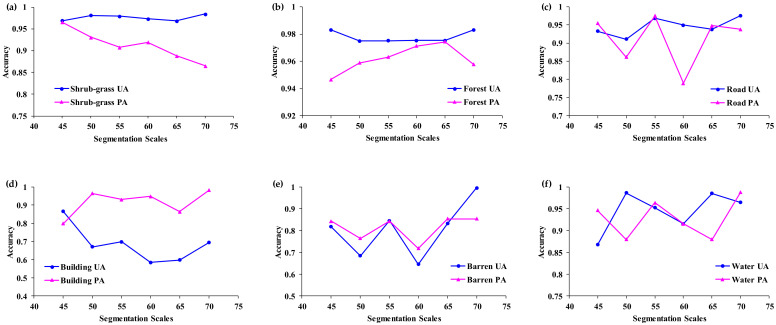
Comparison of the user’s accuracies and producer’s accuracies. (**a**) Shrub-grass; (**b**) Forest; (**c**) Road; (**d**) Building; (**e**) Barren; (**f**) Water.

Comparisons of the shrub-grass and forest’s user’s accuracies and producer’s accuracies are shown in Figure 7. According to the statistical results, the user’s accuracies of the shrub-grass, forest, building, and barren land increase as the segmentation scale increases, but the user’s accuracy of water decreases as the segmentation scale increases. For segmentation scale 70, the user’s accuracies of the shrub-grass, forest, roads, and barren land are the highest at 98.4%, 98.31%, 97.6%, and 99.6%, respectively. The producer’s accuracy of the shrub-grass decreases as the segmentation scale increases. The producer’s accuracy of the forest increases in the scale range of 45–65, but decreases at scale 70. Overall, both the user’s accuracy and producer’s accuracy change with the segmentation scale.

## 4. Discussion

The scale parameter is crucial for the OBIA [27,36,57,58] and the scale selection for high-spatial-resolution images information extraction and processing is important for remote sensing applications [59]. Thus, an optimal scale parameter significantly impacts classification accuracy. In this study, the average areas of land objects were used to evaluate the segmentation scales, and the results indicate that the objects’ average areas reflect the variation characteristics of segmentation scales with the land objects’ areas. In a certain scale range, the classification accuracy was notably affected by changes in the segmentation scale parameters. This was a predictable outcome because similar results have been obtained previously [14,60]. As the remote sensing image’s spatial resolution increased, especially in high-spatial-resolution images, any individual object is modeled by large amounts of pixels [61], which means different scale parameters would obtain different image objects. Therefore, multiscale segmentation is the most important issue for OBIA as there is no single segmentation scale that can represent different image objects. For small scales, a large number of image objects are obtained, and the object polygons are actually over-segmented, which means even a very small land object will be segmented into many individual polygon objects. For instance, in this study, the areas of the building land were smaller than those of the other five types of land objects, and too much redundant information was generated at scale 5, so this was an inadequate scale for building land. For scales 10–25, land objects with small areas and the same texture were generated as individual polygon objects, including shrub-grass land and roads. Even for small scales, very few mixed objects were found in the polygon objects, but most of the objects’ shape were over dissected, and the differences in the object’s spectral and textural features also decreased. This is not applicable to the extraction of land object types, such as forest and shrub-grass land. As the scale increased, the image objects’ areas increased, and some adjacent polygon objects were inevitably merged. This also indicates that the object homogeneity increased and could describe the regional land cover pattern, but it made no sense compared to the accurate image classification. The results described above and showed in Figure 7 show that the UA didn’t decrease regularly as the segmentation scale parameter increased, especially for the shrub-grass, forest, and road; they were relatively stable at a finer segmentation scale parameter. The optimal scale should be equivalent to the size of a specific object, and it should also be able to clearly reveal the boundaries of the different land object polygons after segmentation. For scales 20–30, even very tiny land objects were divided into individual polygon objects, but for scales >50, some of the tiny land objects were merged with an adjacent polygon object to form a new object because their spectral features were too similar. In the process of multiple scale segmentation, not all of the land objects’ areas increased as the segmentation scales increased. Some of the objects’ sizes remained unchanged within a certain scale range. The change in the objects’ sizes or areas was complicated, which means multiple factors affect the image objects’ size, including the segmentation scale, the objects’ spectral and textural features, and the spatial geometric features. Therefore, as the segmentation scale increases, the land object’s ideal size should not be larger than the object’s size in the image. The average areas of land objects exhibited a proper change trend as the segmentation scale increased. This indicates that the optimal segmentation scale for each land object is a continuous scale interval, and it cannot be defined as an absolute value. 

As the segmentation scale increased, the segmented objects’ areas also increased, and the classification accuracies gradually decreased. However, for the segmentation scales selected in this study, the change in the overall accuracy for scales 30–80 tended to be stable. The quality of the segmentation results was affected by multiple factors, including the image data and segmentation parameters. For those land objects that are segmented into the same image object, the purity of the land object can significantly affect the feature extraction and classification rules. As the segmentation scale increases, the image object polygon areas under large segmentation scale are more likely under-segmented, which means these polygons might be misclassified, and a large number of mixed objects would be contained in the object polygon obtained under coarse segmentation scales. The large polygons are not as representative as the polygons obtained under small scale parameters, because the mean spectral values and texture value cannot reflect the real content of the land object [14,62]. A small segmentation scale results in more objects, and the classification accuracies will be higher than for large segmentation scales. However, more objects mean a higher possibility of redundancy in the information extraction, and the same land object will be probably be classified into different categories. Overall, selecting an optimal segmentation scale is the most important part of the OBIA, but since the spectral and textural features of different land objects are different, their spatial and geometric features are also different, so the optimal segmentation scales of different land objects are not the same. Thus, a targeted segmentation scale for corresponding land objects is important. 

## 5. Conclusions

The goal of this study was to investigate the effect of the segmentation scale on classification results and accuracies. In this study, different segmentation scales were used in the OBIA to investigate the segmentation scale effect on WV-3 image classification. Since different land objects’ have different spectral and textural features, a single segmentation scale may not be the best choice for image classification. Since different land objects might show similarities in both spectral and texture characteristics, it is difficult to identify them at the same scale, and it is obvious that different land objects correspond to different optimal segmentation scales. Therefore, to extract hierarchical information with good accuracy, the selection of optimal segmentation scale is a prerequisite [63]. Based on the classification results of different segmentation results, an equation between the land object average areas and segmentation scales was established. The correlation coefficient between the land objects’ average areas and their optimal segmentation scales is 0.847, and it demonstrated that the land object’s average areas were proportional to the optimal segmentation scale. The results of this study show that the land objects’ average areas are significantly correlated to their optimal segmentation scales, which can help determine the optimal segmentation scale for the corresponding objects. It is no doubt that the segmentation scale parameter impacts classification accuracy. Our results showed that the OBIA method is a high efficiency tool for high-spatial resolution image; the average area-based method presented in this study can be useful for optimal segmentation scale selection, especially when the land objects vary in size and shape. However, some research focuses on other aspects to achieve higher accuracy [14,38,64,65], like the influence of training set size and classification method. Thus, selecting an optimal parameter for image segmentation would never be the only solution for obtaining higher classification accuracy in OBIA. In addition, potential limitations of this study should be considered and studied in future research. For future works, we also intend to evaluate and compare the performance of the CART with other machine learning classifiers and deep learning-based methods in different sensor images of long time series. 

## Figures and Tables

**Table 1:** Selected spectral and vegetation indices for the CART decision tree rulesets.

Features		Formula	Reference or Note
Spectral Bands	B1	$\frac{1}{n}\sum_{\left(x,y\right)\in P_{v}}C_{k}\left(x,y\right),$ $C_{k}\left(x,y\right)$ is spectral value of pixel (*x*, *y*), *n* is pixel’s total amount, *k* is the spectral band	CoastalBlue (427 nm) Blue (482 nm) Green (547 nm) Yellow (604 nm) Red (660 nm) RedEdge (723 nm) NIR1 (824 nm) NIR2 (914 nm)
	B2		
	B3		
	B4		
	B5		
	B6		
	B7		
	B8		
	Brightness	$\frac{1}{n_{L}}\sum_{i=1}^{n_{L}}{\bar{c}}_{i}\left(v\right)$	${\bar{c}}_{i}\left(v\right)$ is object v’s brightness, ${\bar{c}}_{i}\left(v\right)$$,\;{\bar{c}}_{j}\left(v\right)$ is the average band value of object *v* in band *i* and band *j*, *n_L_* is band’s total amount [40]
	Max Difference	$\frac{\underset{i,j\in K_{B}}{\max}\left|{\bar{c}}_{i}\left(v\right)-{\bar{c}}_{j}\left(v\right)\right|}{\bar{c}\left(v\right)}$	
Vegetation Indices	NDVI	(*NIR* − *R*)/(*NIR* + *R*)	R: Red band; G: Green Band; NIR: Near-infrared band [42,43,44,45]
	RVI	*NIR*/*R*	
	DVI	*NIR*-*R*	
	NDWI	(*G* − *NIR*)/(*G* + *NIR*)	

**Table 2:** Textural features for the CART decision tree rulesets.

GLCM Texture	Formula	Reference or Note
Mean	$\frac{\sum_{i,j=0}^{N-1}P(i,j)}{N^{2}}$	(1) *P*(*i*, *j*) is the normalized co-occurrence matrix, the calculation equation can be expressed as: $P\left(i,\;j\right)=\frac{C_{i,j}}{\sum_{i,j=0}^{N-1}C_{i,j}}$ (2) *ME* is the mean value of the gray level co-occurrence matrix. (3) *N* stands for the columns or lines of the statistical matrix. *C_i_*_,*j*_ stands for the (*i*, *j*) unit value of the statistical matrix [40,49,50]
Variance	$\sum_{i,j=0}^{N-1}P\left(i,\;j\right){\left(1-ME\right)}^{2}$	
Homogeneity	$\sum_{i,j=0}^{N-1}i\frac{P\left(i,j\right)}{1+{\left(i-j\right)}^{2}}$	
Contrast	$\sum_{i,j=0}^{N-1}P(i,j){\left(i-j\right)}^{2}$	
Dissimilarity	$\sum_{i,j=0}^{N-1}P(i,j)\left|i-j\right|$	
Entropy	$\sum_{i,j=0}^{N-1}P(i,j)(-\ln P_{i,j})$	
Secondary Moment	$\sum_{i,j=0}^{N-1}iP{\left(i,j\right)}^{2}$	

**Table 3:** Evaluating indicator values for the validation of the CART model.

Segmentation Scale	AUC	Precision	Recall	OA
45	0.9455	0.9569	0.8636	0.9338
50	0.9240	0.8672	0.8185	0.9054
55	0.9199	0.7699	0.7379	0.9362
60	0.9373	0.9001	0.8488	0.8882
65	0.9704	0.9406	0.9479	0.9178
70	0.9182	0.7099	0.7132	0.9210
